# Substituted *N-*Benzylpyrazine-2-carboxamides: Synthesis and Biological Evaluation ^†^

**DOI:** 10.3390/molecules171113183

**Published:** 2012-11-06

**Authors:** Barbora Servusová, Drahomíra Eibinová, Martin Doležal, Vladimír Kubíček, Pavla Paterová, Matúš Peško, Katarína Kráľová

**Affiliations:** 1 Department of Pharmaceutical Chemistry and Drug Control, Faculty of Pharmacy, Charles University in Prague, Heyrovského 1203, 500 05 Hradec Králové, Czech Republic; Email: eibid5aa@faf.cuni.cz (D.E.); dolezalm@faf.cuni.cz (M.D.); 2 Department of Biophysics and Physical Chemistry, Faculty of Pharmacy, Charles University in Prague, Heyrovského 1203, 500 05 Hradec Králové, Czech Republic; Email: kubicek@faf.cuni.cz; 3 Department of Clinical Microbiology, University Hospital, Sokolská 581, 500 05 Hradec Králové, Czech Republic; Email: pavla.paterova@fnhk.cz; 4 Department of Ecosozology and Physiotactics, Faculty of Natural Sciences, Comenius University, Mlynská Dolina Ch-2, 84215 Bratislava, Slovakia; Email: matus.pesko@gmail.com; 5 Institute of Chemistry, Faculty of Natural Sciences, Comenius University, Mlynská Dolina CH-2, 842 15 Bratislava, Slovakia; Email: kralova@fns.uniba.sk

**Keywords:** pyrazinamide analogues, lipophilicity determination, *in vitro* antimycobacterial, antifungal and photosynthesis inhibitory activity

## Abstract

A series of twelve amides was synthesized *via* aminolysis of substituted pyrazinecarboxylic acid chlorides with substituted benzylamines. Compounds were characterized with analytical data and assayed *in vitro* for their antimycobacterial, antifungal, antibacterial and photosynthesis-inhibiting activity. 5-*tert*-Butyl-6-chloro-*N*-(4-methoxybenzyl)pyrazine-2-carboxamide (**12**) has shown the highest antimycobacterial activity against *Mycobacterium tuberculosis* (MIC = 6.25 µg/mL), as well as against other mycobacterial strains. The highest antifungal activity against *Trichophyton mentagrophytes*, the most susceptible fungal strain tested, was found for 5-chloro-*N*-(3-trifluoromethylbenzyl)-pyrazine-2-carboxamide (**2**, MIC = 15.62 µmol/L). None of the studied compounds exhibited any activity against the tested bacterial strains. Except for 5-*tert*-butyl-6-chloro-*N*-benzylpyrazine-2-carboxamide (**9**, IC_50_ = 7.4 µmol/L) and 5-*tert*-butyl-6-chloro-*N*-(4-chlorobenzyl)pyrazine-2-carboxamide (**11**, IC_50_ = 13.4 µmol/L), only moderate or weak photosynthesis-inhibiting activity in spinach chloroplasts (*Spinacia oleracea* L.) was detected.

## 1. Introduction

Tuberculosis (TB) is considered to be one of the most frequent and widespread nowadays infectious diseases especially in developing countries. In 2010, there were about 8.8 million new cases of TB and 1.4 million deaths (including deaths from TB among HIV-positive people) [1]. Anti-TB drug resistance is a major public health problem that threatens progress made in TB care and control worldwide. Particularly dangerous forms of TB are multidrug-resistant TB (MDR-TB) and extensively drug-resistant TB (XDR-TB) [1]. Furthermore, TB and HIV synergistically influence each other’s progress and lead to the increased need of new antituberculars [2]. 

Pyrazinamide (PZA), a nicotinamide analogue, is one of the most important first-line drugs used in TB-therapy [3]. Along with rifampicin, PZA has sterilizing activity (the ability to kill the semi-dormant mycobacteria) which is a crucial factor in shortening the duration of therapy [4]. PZA, as a prodrug that requires bacterial enzymes to generate the biologically active molecule, enters mycobacterial cell via passive diffusion and it is activated by pyrazinamidase/nicotinamidase (EC 3.5.1.19) to form pyrazinoic acid (POA) [5]. Pyrazinamidase/nicotinamidase is encoded by the *pncA* gene and mutation of this gene is primarily responsible for resistance to PZA [6]. POA’s intracellular accumulation lowers pH in mycobacterial cell, thus leading to inhibition of membrane transport and depletion of energy [7]. Otherwise, the demonstration that PZA and POA inhibit *Mycobacterium tuberculosis* fatty acid synthase-I (FAS-I) in whole-cell and cell-free assays suggests that the disruption might be a consequence of the inhibition of membrane synthesis [8,9,10]. Reversible binding of both PZA and POA to *M. tuberculosis* FAS-I has been definitively confirmed by Saturation Transfer Difference NMR spectroscopy (STD-NMR), a NMR technique used to characterize ligand–protein interactions [11]. Boshoff *et al.* [12] reported that FAS-I is not the target of PZA. However, FAS-I has been proposed and confirmed as a target of pyrazinamide derivatives, e.g., 5-chloropyrazinamide [8,9]. Finally, another specific target for POA, ribosomal protein S1 (RpsA), has been identified in recent study [13]. RpsA is a vital protein involved in protein translation and the ribosome-sparing process of *trans*-translation and its role in *M. tuberculosis* is multifaceted. 

Several pyrazine derivatives were found to possess herbicidal activity summarized in review paper [14]. Many pyrazinamide derivatives inhibited photosynthetic electron transport (PET) in plant chloroplasts [14,15,16,17] and they were found to act as photosystem (PS) 2 inhibitors. Using EPR spectroscopy it was found that 5-*tert*-butyl-*N*-(3-hydroxy-4-chlorophenyl)-pyrazine-2-carboxamide and 5-*tert*-butyl-6–chloro-*N*-(3-fluorophenyl)-pyrazine-2-carboxamide interacted with the D^·^ intermediate, *i.e.*, with the tyrosine radical which is situated at 161th position on D_2_ protein occurring on the donor side of PS 2 [18]. Due to this interaction, PET from the oxygen evolving complex to the reaction centre of PS 2 is impaired. However, an experiment with artificial electron donor 1,5-diphenylcarbazide (DPC) with known site of action in Z^·^/D^·^ intermediate confirmed that also some members of the PET chain between Z^·^/D^·^ intermediate and plastoquninone were partially damaged in the light by these carboxamides. Chlorophyll *a* fluorescence quenching due to treatment of chloroplast suspension with above mentioned pyrazinamides indicated their interaction with pigment-protein complexes in PS 2 [18].

In this study, we focused on binuclear pyrazinamide analogues containing the -CONH-CH_2_- bridge, namely on *N*-benzylpyrazine-2-carboxamides. Earlier studies have shown some interesting anti-mycobacterial activity in a series of substituted *N*-phenylpyrazine-2-carboxamides [16,19] and become a pattern for substitution of aromatic ring in benzylamines. The aim of this work was to find the structure-activity relationships (SAR) in the series of substituted *N*-benzylpyrazine-2-carboxamides, *i.e.*, to study the influence of incorporated methylene moiety in the connecting bridge and to continue in the study of the substituent variability influence on the biological activity. 

## 2. Results and Discussion

### 2.1. Chemistry

**Scheme 1:**
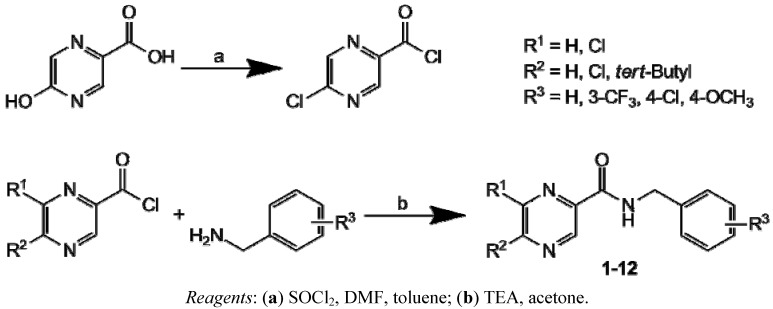
Synthesis and structure of the 5-chloropyrazine-2-carbonyl chloride and final products **1**–**12**.

Synthesis of 5-chloropyrazine-2-carboxylic acid chloride [20] from 5-hydroxypyrazine-2-carboxylic acid, as well as synthesis of final compounds **1**–**12**, is shown in Scheme 1. Condensation of chlorides of 5-chloropyrazine-2-carboxylic [20], 6-chloropyrazine-2-carboxylic [21] and 5-*tert-*butyl-6-chloropyrazine-2-carboxylic [22] acids with unsubstituted or ring-substituted benzylamines yielded a series of twelve amides. Reactions proceeded under mild conditions, yields of products ranged within 59–91%, and analytical data were fully consistent with the proposed structures. Specific substituents R^1^, R^2^ and R^3^ of individual compounds **1**–**12** are listed in Table 1.

**Table 1:** Comparison of the calculated lipophilicity (log *P*, Clog *P*) with the determined log *k* values of the studied compounds **1**–**12**. IC_50_ values related to PET inhibition in spinach chloroplasts in comparison with the standard 3-(3,4-dichlorophenyl)-1,1-dimethylurea (DCMU).

Compounds	R^1^	R^2^	R^3^	log *P*	Clog *P*	log *k*	IC_50_ [µmol/L]
1	H	Cl	H	1.56	2.3852	0.1862	1623.0
2	H	Cl	3-CF_3_	2.48	3.2682	0.4384	345.8
3	H	Cl	4-Cl	2.12	3.0982	0.4150	604.0
4	H	Cl	4-OCH_3_	1.43	2.3042	0.1655	ND
5	Cl	H	H	1.56	2.3852	0.2002	ND
6	Cl	H	3-CF_3_	2.48	3.2682	0.4507	1207.0
7	Cl	H	4-Cl	2.12	3.0982	0.4335	ND
8	Cl	H	4-OCH_3_	1.43	2.3042	0.1838	ND
9	Cl	(CH_3_)_3_C	H	3.69	4.2112	1.1215	7.4
10	Cl	(CH_3_)_3_C	3-CF_3_	4.61	5.0942	1.3638	36.3
11	Cl	(CH_3_)_3_C	4-Cl	4.25	4.9242	1.3511	13.4
12	Cl	(CH_3_)_3_C	4-OCH_3_	3.56	4.1302	0.8071	121.6
DCMU	–	–	–	–	–	–	1.9

ND not determined due to their low solubility in the testing medium.

### 2.2. Lipophilicity

**Figure 1:**
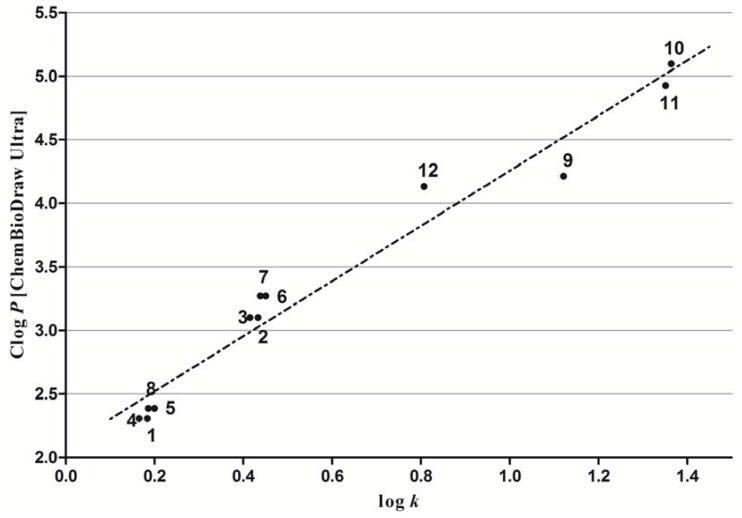
Plot of calculated Clog *P* (CS ChemBioDraw Ultra version 12.0) on experimentally measured log *k* parameter. Linear regression parameters.

Lipophilicity, one of the most important physicochemical properties of the compound, which seems to be a key factor related to the cell transmembrane transport and other biological processes, can either be determined experimentally or predicted by means of commercially available programmes. In this work Log *P*/Clog *P* values of the compounds **1**–**12** were calculated using the program CS ChemBioDraw Ultra version 12.0 (CambridgeSoft, Cambridge, MA, USA) and also measured by means of the RP-HPLC determination of capacity factors *k* with subsequent calculation of log *k*. The results are shown in Table 1 and illustrated in Figure 1. 

The lowest lipophilicity was shown by 5-chloro-*N*-(4-methoxybenzyl)pyrazine-2-carboxamide (**4**), while 5-*tert*-butyl-6-chloro-*N*-(3-trifluoromethylbenzyl)pyrazine-2-carboxamide (**10**) was the most lipophilic compound of this series. Based on log *k* values, lipophilicity increased for substituents in pyrazine part in the following order: 5-chloropyrazine < 6-chloropyrazine < 5-*tert*-butyl-6-chloropyrazine. In the case of substituents in the benzyl part of the molecule lipophilicity increased this way: 4-OCH_3_ < H < 4-Cl < 4-CF_3_. The dependence of the calculated Clog *P* values on the measured log *k* parameters showed an approximate linearity and the corresponding correlation can be expressed by the following regression equation:

Clog *P* = 2.170 (±0.131) log *k* + 2.086 (±0.096) (1)

r = 0.9650 s = 0.197 F = 276.1 n = 12

The differences between experimentally determined log *k* and calculated Clog *P* values were observed for 5-chloro and 6-chloro group in pyrazine part. This may be caused by the used calculating program for Clog *P*, that does not distinguish difference between substituent’s lipophilicity in position C(5) and C(6) in pyrazine part of molecule. Consequently, it can be assumed that log *k* values specify lipophilicity within this series of compounds more precisely than calculated Clog *P* values.

### 2.3. Biological Activity

#### 2.3.1. *In Vitro* Antimycobacterial Evaluation

All synthesized compounds were assayed *in vitro* towards *Mycobacterium tuberculosis* and several Mycobacteria Other Than Tuberculosis (MOTTs) (see Table 2). The most active derivatives against *M. tuberculosis* were compounds **8**, **10** and **12**, whose minimal inhibition concentrations (MICs) were 6.25 µg/mL. These activities were fully comparable with PZA. More importantly, 5-*tert*-butyl-6-chloro-*N*-(4-methoxybenzyl)pyrazine-2-carboxamide (**12**) showed significant activity against tested MOTTs, which are unsusceptible to PZA. The vast majority of compounds exhibited only modest antimycobacterial activity expressed as MIC in µg/mL, or with respect to the molecular weight of final products in µmol/L (see Table 2). 

The obtained results provide some insights into the SAR in this miniseries. With respect to the benzyl part, the most suitable substituents are 4-methoxy and 3-trifluoromethyl groups, especially with disubstitution in the pyrazine moiety. The most significant substitutions in the pyrazine part are chlorine in the C(6) and *tert-*butyl in the C(5) position. 

There is no clear dependence between lipophilicity (log *k*, see Table 1) and antimycobacterial activities in this series, however the most lipophilic compound 5-*tert*-butyl-6-chloro-*N*-(3-trifluoromethylbenzyl)pyrazine-2-carboxamide (**10**, log *k* = 1.3638) displayed one of the highest activity against *M. tuberculosis*. On the other hand, 6-chloro-*N*-(4-methoxybenzyl)pyrazine-2-carboxamide (**8**) with the lowest lipophilicity (log *k* = 0.1838) showed the same activity against *M. tuberculosis* as compound **10** (MIC = 6.25 µg/mL). 

**Table 2:** Antimycobacterial and antifungal activity of presented compounds in comparison with standards: pyrazinamide (PZA), isoniazid (INH) and fluconazole (FLU).

Compounds	MIC [µg/mL]				TM ^e^ MIC [µmol/L]
	*M. tuberculosis* H37Rv ^a^	*M. avium* ^b^	*M. avium* ^c^	*M. kansasii* ^d^	
1	25 (100)	100	100	100	125/125
2	25 (79)	25	100	50	15.62/15.62
3	12.5 (44)	100	100	100	125/125
4	25 (90)	100	50	100	250/250
5	12.5 (50)	100	100	50	250/500
6	12.5 (39)	25	100	100	62.5/125
7	12.5 (44)	100	12.5	100	125/125
8	6.25 (22)	100	50	100	500/500
9	25 (82)	25	50	100	250/500
10	6.25 (16)	25	25	25	250/250
11	12.5 (36)	50	50	50	250/500
12	6.25 (18)	12.5	6.25	3.125	250/500
PZA	6.25–12.5 (50–101)	>100	>100	>100	–
INH	1.56 (11)	12.5–25	12.5	12.5	–
FLU	–	–	–	–	1.95/3.91

^a^ CNCTC My 331/88; ^b^ CNCTC My 80/72; ^c^ CNCTC My 152/73; ^d^ CNCTC My 235/80; ^e^ TM *Trichophyton mentagrophytes* 445, evaluated after 72 h/120 h.

#### 2.3.2. *In Vitro* Antifungal Evaluation

The evaluation of *in vitro* antifungal activity of the studied compounds was performed against eight fungal strains. Except for 5-chloro- (**2**) and 6-chloro-*N*-(3-trifluoromethylbenzyl)pyrazine-2-carboxamides (**6**), only weak antifungal activity was found. In comparison with fluconazole (the standard, MIC = 1.95 µmol/L after 72 h) compounds **2** (MIC = 15.62 µmol/L) and **6** (MIC = 62.5 µmol/L) exhibited moderate *in vitro* antifungal activity against *Trichophyton mentagrophytes* (TM), the most susceptible fungal strain evaluated. For the results see Table 2.

#### 2.3.3. *In Vitro* Antibacterial Evaluation

All prepared compounds were tested for their *in vitro* antibacterial activity [23,24] against eight bacterial strains, namely against: *Staphylococcus aureus* CCM 4516/08, *Staphylococcus aureus* H 5996/08–methicilin resistant, *Staphylococcus epidermidis* H 6966/08, *Pseudomonas aeruginosa* CCM 1961, *Escherichia coli* CCM 4517. *Enterococcus sp.* J 14365/08, *Klebsiella pneumoniae* D 11750/08 and *Klebsiella pneumoniae* J 14368/08–ESBL positive. None of the synthesized compounds exhibited any activity against the tested strains. 

#### 2.3.4. Inhibition of Photosynthetic Electron Transport (PET)

All studied compounds were evaluated for their photosynthetic electron transport (PET) inhibition in spinach chloroplasts, which was reflected in the inhibition of oxygen evolution rate. The photosynthesis-inhibiting activity of the compounds has been expressed as IC_50_ values. Compounds **4**, **5**, **7** and **8 **were not tested due to their low solubility in tested medium. The IC_50_ values varied in the range from 7.4 to 1,623.0 µmol/L, see Table 1. The activity of the majority of compounds was moderate or relatively low when compared with the standard 3-(3,4-dichlorophenyl)-1,1-dimethylurea (DCMU, IC_50_ = 1.9 µmol/L).

**Figure 2:**
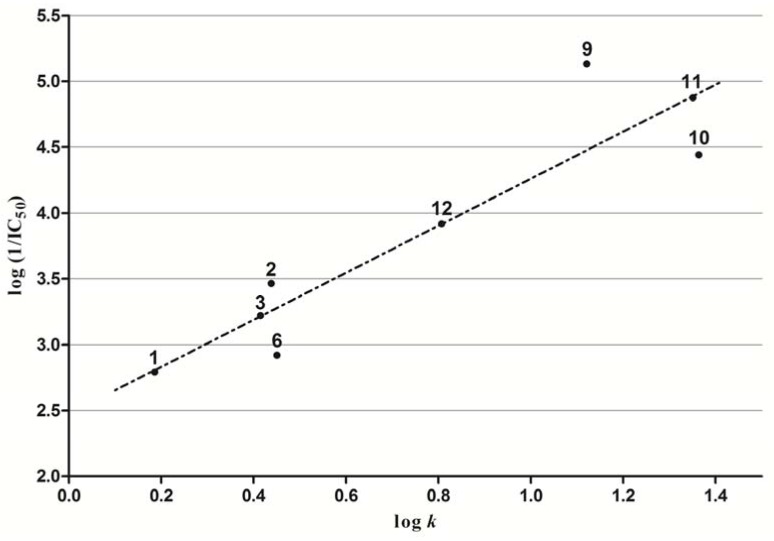
Linear dependence between lipophilicity (expresed as logarithm of retention factor, log *k*) and photosynthesis-inhibiting activity log (1/IC_50_) [mol/L]. of synthesized compounds **1**–**12**.

The most efficient inhibitors of this series were compounds **9** (IC_50_ = 7.4 µmol/L) and **11** (IC_50_ = 13.4 µmol/L). In general, photosynthesis-inhibiting activity of the studied compounds is dependent on their lipophilicity (compare compounds **9**–**12**, Table 1). PET inhibition of compounds **1**–**12** has increased linearly with increasing lipophilicity expressed as log *P* or log *k* (see Figure 2) and the corresponding correlations can be expressed by the following regression equations:

log (1/IC_50_) = 1.626 (±0.534) + 0.717 (±0.164) log *P* (2)

r = 0.8722 s = 0.472 F = 19.08 n = 8

log (1/IC_50_) = 2.475 (±0.266) +1.785 (±0.303) log *k* (3)

r = 0.92358 s= 0.370 F = 34.82 n = 8

The results of statistical analysis were improved if log *k* instead log *P* was used. This finding is in agreement with above mentioned assumption that log *k* values specify lipophilicity within this series of compounds more precisely than calculated log *P* values. Log *k* values of the most active inhibitors were about 1.2.

**Figure 3:**
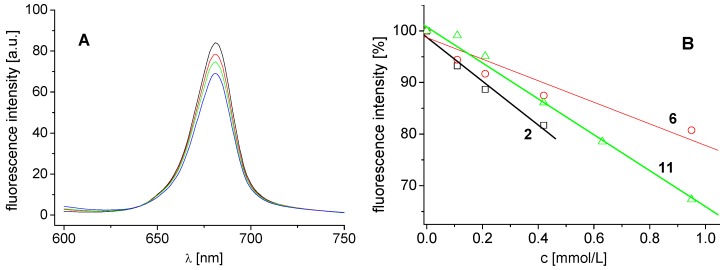
(**A**) Fluorescence emission spectra of chlorophyll *a* in untreated spinach chloroplasts in the presence of compound **2**: 0, 0.11, 0.21 and 0.42 mmol/L (curves from top to bottom; λ_ex_ = 436 nm). (**B**) Dependence of fluorescence intensity of chlorophyll *a* on concentration of compounds **2** (squares), **11** (triangles) and **6** (circles).

The effects of the studied compounds on the photosynthetic apparatus of spinach chloroplasts were investigated by studying chlorophyll *a* (Chl*a*) fluorescence. Fluorescence emission spectra of Chl*a* in spinach chloroplasts treated with compound **2** are shown in Figure 3A. The decreased intensity of the emission band at 686 nm belonging to the pigment-protein complexes in photosystem 2 [25] suggested PS 2 as the site of action of the studied inhibitors. The extent of perturbation of chlorophyll *a*-protein complexes in the thylakoid membrane was reflected as decreased fluorescence of the pigment (see Figure 3B). Similar decrease of Chl*a* fluorescence in plant chloroplasts was also observed previously after treatment with substituted benzanilides [26] and salicyanilides [27]. 

**Figure 4:**
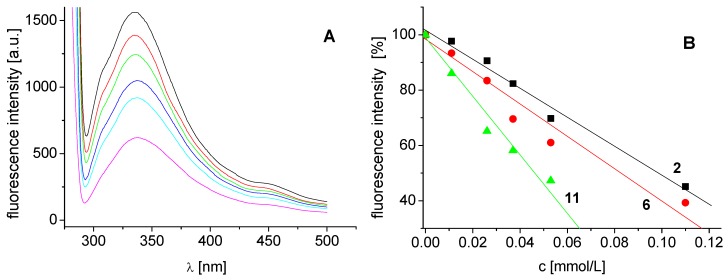
(**A**) Fluorescence emission spectra of aromatic amino acids in untreated spinach chloroplasts in presence of compound **6**: 0, 0.011, 0.026, 0.037, 0.053 and 0.11 mmol/L(curves from top to bottom; λ_ex_ = 275 nm). (**B**) Dependence of fluorescence intensity of aromatic amino acids on concentration of compounds **2** (squares), **11** (triangles) and **6** (circles).

Interaction of the studied compounds with aromatic amino acids, which are present in the proteins of spinach chloroplasts situated in PS 2, was documented by the quenching of their fluorescence at 334 nm. Figure 4 presents fluorescence emission spectra of aromatic amino acids of untreated spinach chloroplasts and of chloroplasts treated with increasing concentrations of compound 6 (see Figure 4A) as well as dependence of fluorescence intensity of chlorophyll *a* on concentration of compounds **2** (squares), **11** (triangles) and **6** (circles) (see Figure 4B). Binding of these compounds to aromatic amino acids occurring in photosynthetic proteins contribute to PET inhibition. 

By the addition of DPC, an artificial electron donor acting in Z^·^/D^·^ intermediate on the donor side of PS 2, to chloroplasts treated with the studied compounds in which PET was inhibited at about 80‑90%, PET was restored only to 77‑88%. This indicates that the site of PET inhibition is situated not only on the donor side of PS 2 in the section between the primary electron donor of PS 2 (H_2_O) and Z^·^/D^·^ intermediate but also in the photosynthetic transport chain from P 680 to plastoquinone Q_B_ occurring on the acceptor side of PS 2. Similar sites of action were determined previously for 5-*tert*-butyl-*N*-(3-hydroxy-4-chlorophenyl)-pyrazine-2-carboxamide and 5-*tert*-butyl-6–chloro-*N*-(3-fluorophenyl)-pyrazine-2-carboxamide [18].

## 3. Experimental

### 3.1. General

All organic solvents used for the synthesis were of analytical grade. All chemicals were purchased from Sigma-Aldrich (Schnelldorf, Germany). The reactions were monitored using Merck Silica 60 F_254_ TLC plates (Merck, Darmstadt, Germany). Compounds were purified using an automated chromatograph CombiFlash *R*_f_ (Teledyne Isco, Lincoln, NE, USA) using columns filled with Kieselgel 60, 0.040–0.063 mm (Merck, Darmstadt, Germany); gradient elution (hexane/ethyl-acetate), detection wavelength 260 nm, monitor wavelength 280 nm. NMR analysis was performed on Varian Mercury VX-BB 300 (Varian, Palo Alto, CA, USA) at 300 MHz for ^1^H and 75 MHz for ^13^C. Chemical shifts were recorded as δ values in parts per million (ppm) and were indirectly referenced to tetramethylsilane (TMS). IR spectra were recorded in KBr blocks on Nicolet Impact 400 (Nicolet, Madison, WI, USA). Elementary analysis was performed on CE Instruments EA-1110 CHN analyser (CE Instruments, Wigan, UK). Melting points were determined on Stuart SMP30 melting point apparatus (Bibby Scientific Limited, Staffordshire, UK) and are uncorrected.

### 3.2. Synthesis of N-Benzylpyrazine-2-carboxamides

To a solution of 6-chloropyrazine-2-carboxylic [21] or 5-*tert-*butyl-6-chloropyrazine-2-carboxylic [22] acid (0.5 mmol) in dry toluene (20 mL) was added 1.5 equivalent of thionyl chloride (0.75 mmol). The reaction mixture was heated to reflux for about 1 h. Then, the excess of thionyl chloride was removed by repeated evaporation with dry toluene *in vacuo*. The crude acyl chloride was dissolved in dry acetone (20 mL) and added dropwise to a stirred solution of the corresponding benzylamine (0.5 mmol) with triethylamine (0.5 mmol) in dry acetone (10 mL). Then, the reaction mixture was stirred at room temperature for about 1 h. The reaction was monitored using TLC with hexane/ethyl acetate 2:1 mixture as eluent. After this time, the solution was evaporated till dryness with silica gel and purified using a flash column chromatography (40 g column, gradient elution hexane/ethyl-acetate).

In the case of 5-chloropyrazine-2-carbonyl chloride [20] synthesis, 5-hydroxypyrazine-2-carboxylic acid (Sigma-Aldrich) was used as starting material. During the reaction with thionyl chloride, the formation of acyl chloride occurs simultaneously with the nucleophilic substitution of the hydroxyl group for chlorine. Dimethylformamide (DMF) was added to the reaction mixture as catalyst [28].

### 3.3. Data of Prepared Target Compounds

*N-Benzyl-5-chloropyrazine-2-carboxamide* (**1**). White crystalline compound. Yield: 81%; m.p. 101.9–103.1 °C; ^1^H-NMR (CDCl_3_) δ 9.21 (s, 1H, H3), 8.49 (s, 1H, H6), 7.98 (bs, 1H, NH), 7.40–7.26 (m, 5H, H2′, H3′, H4′, H5′, H6′), 4.67 (d, 2H, *J* = 6.0 Hz, NCH_2_); ^13^C-NMR (CDCl_3_) δ 162.0, 152.1, 144.0, 142.6, 142.4, 137.5, 128.8, 127.8, 127.7, 43.5; IR (cm^−1^) 3384 (N-H), 1660 (C=O); Anal. Calcd. For C_12_H_10_ClN_3_O (247.68): 58.19% C, 4.07% H, 16.97% N; Found: 58.38% C, 4.23% H, 16.89% N.

*5-Chloro-N-(3-trifluoromethylbenzyl)pyrazine-2-carboxamide* (**2**). White crystalline compound. Yield: 65%; m.p. 91.3–92.7 °C; ^1^H-NMR (CDCl_3_) δ 9.20 (s, 1H, H3), 8.51 (s, 1H, H6), 8.09 (bs, 1H, NH), 7.65–7.40 (m, 4H, H2′, H4′, H5′, H6′), 4.72 (d, 2H, *J* = 6.3 Hz, NCH_2_); ^13^C-NMR (CDCl_3_) δ 162.2, 152.3, 144.0, 142.5, 142.3, 138.6, 131.2, 131.1 (q, *J* = 32.6 Hz), 129.3, 124.6 (q, *J* = 3.8 Hz), 124.4 (q, *J* = 3.8 Hz), 123.6 (q, *J* = 272.9 Hz), 43.0; IR (cm^−1^) 3385 (N-H), 1665 (C=O); Anal. Calcd. For C_13_H_9_ClF_3_N_3_O (315.68): 49.46% C, 2.87% H, 13.31% N; Found: 49.51% C, 2.96% H, 13.48% N.

*5-Chloro-N-(4-chlorobenzyl)pyrazine-2-carboxamide* (**3**). White crystalline compound. Yield: 79%; m.p. 89.9–91.3 °C; ^1^H-NMR (CDCl_3_) δ 9.20 (s, 1H, H3), 8.50 (s, 1H, H6), 7.98 (bs, 1H, NH), 7.35–7.25 (m, 4H, H2′, H3′, H5′, H6′), 4.63 (d, 2H, *J* = 6.2 Hz, NCH_2_); ^13^C-NMR (CDCl_3_) δ 162.1, 152.2, 144.0, 142.5, 142.4, 136.1, 133.6, 129.2, 128.9, 42.9; IR (cm^−1^) 3327 (N-H), 1672 (C=O); Anal. Calcd. For C_12_H_9_Cl_2_N_3_O (282.13): 51.09 % C, 3.22% H, 14.89% N; Found: 50.96% C, 3.14% H, 14.97% N.

*5-Chloro-N-(4-methoxybenzyl)pyrazine-2-carboxamide* (**4**). White crystalline compound. Yield: 80%; m.p. 123.2–124.0 °C; ^1^H-NMR (CDCl_3_) δ 9.19 (s, 1H, H3), 8.48 (s, 1H, H6), 7.90 (bs, 1H, NH), 7.30–7.25 (m, 2H, H2′, H6′), 6.99–6.62 (m, 2H, H3′, H5′), 4.59 (d, 2H, *J* = 5.9 Hz, NCH_2_), 3.80 (s, 3H, CH_3_); ^13^C-NMR (CDCl_3_) δ 161.9, 159.2, 152.0, 144.0, 142.6, 142.4, 129.6, 129.3, 114.2, 55.3, 43.1; IR (cm^−1^) 3388 (N-H), 1659 (C=O); Anal. Calcd. For C_13_H_12_ClN_3_O_2_ (277.71): 56.22% C, 4.36% H, 15.13% N; Found: 56.26% C, 4.34% H, 15.01% N.

*N-Benzyl-6-chloropyrazine-2-carboxamide* (**5**). White crystalline compound. Yield: 87%; m.p. 58.3–59.7 °C; ^1^H-NMR (DMSO-*d_6_*) δ 9.48 (t, 1H, *J* = 6.3 Hz, NH), 9.19 (s, 1H, H3), 8.81 (s, 1H, H5), 7.34–7.20 (m, 5H, H2′, H3′, H4′, H5′, H6′), 4.50 (d, 2H, *J* = 6.3 Hz, NCH_2_); ^13^C-NMR (DMSO-*d_6_*) δ 163.1, 148.1, 143.8, 143.6, 143.1, 139.4, 128.4, 127.5, 127.0, 42.5; IR (cm^−1^) 3371 (N-H), 1670 (C=O); Anal. Calcd. For C_12_H_10_ClN_3_O (247.68): 58.19% C, 4.07% H, 16.97% N; Found: 58.33% C, 4.12% H, 17.08% N.

*6-Chloro-N-(3-trifluoromethylbenzyl)pyrazine-2-carboxamide* (**6**). White crystalline compound. Yield: 63%; m.p. 68.1–69.8 °C; ^1^H-NMR (CDCl_3_) δ 9.33 (s, 1H, H3), 8.77 (s, 1H, H5), 8.03 (bs, 1H, NH), 7.64–7.42 (m, 4H, H2′, H4′, H5′, H6′), 4.73 (d, 2H, *J* = 6.3 Hz, NCH_2_); ^13^C-NMR (CDCl_3_) δ 161.8, 147.6, 147.5, 143.6, 142.1, 138.5, 131.2 (q, *J* = 32.4 Hz), 130.9, 129.3, 124.6 (q, *J* = 3.9 Hz), 124.5 (q, *J* = 4.0 Hz), 124.0 (q, *J* = 272.9 Hz), 43.1; IR (cm^−1^) 3324 (N-H), 1672 (C=O); Anal. Calcd. For C_13_H_9_ClF_3_N_3_O (315.68): 49.46% C, 2.87% H, 13.31% N; Found: 49.57% C, 2.71% H, 13.56% N.

*6-Chloro-N-(4-chlorobenzyl)pyrazine-2-carboxamide* (**7**). White crystalline compound. Yield: 91%; m.p. 93.2–94.5 °C; ^1^H-NMR (CDCl_3_) δ 9.32 (s, 1H, H3), 8.76 (s, 1H, H5), 7.97 (bs, 1H, NH), 7.37–7.31 (m, 2H, H2′, H6′), 7.28–7.20 (m, 2H, H3′, H5′), 4.63 (d, 2H, *J* = 6.1 Hz, NCH_2_); ^13^C-NMR (CDCl_3_) δ 161.7, 147.5, 147.4, 143.7, 142.0, 135.9, 133.6, 129.2, 128.9, 42.9; IR (cm^−1^) 3387 (N-H), 1667 (C=O); Anal. Calcd. For C_12_H_9_Cl_2_N_3_O (282.13): 51.09% C, 3.22% H, 14.89% N; Found: 51.23% C, 3.37% H, 14.76% N.

*6-Chloro-N-(4-methoxybenzyl)pyrazine-2-carboxamide* (**8**). White crystalline compound. Yield: 89%; m.p. 71.4–72.6 °C; ^1^H-NMR (CDCl_3_) δ 9.32 (s, 1H, H3), 8.73 (s, 1H, H5), 7.88 (bs, 1H, NH), 7.31–7.25 (m, 2H, H2′, H6′), 6.91–6.85 (m, 2H, H3′, H5′), 4.59 (d, 2H, *J* = 6.0 Hz, NCH_2_), 3.80 (s, 3H, CH_3_); ^13^C-NMR (CDCl_3_) δ 161.5, 159.2, 147.5, 147.2, 144.0, 142.0, 129.5, 129.3, 114.2, 55.3, 43.1; IR (cm^−1^) 3370 (N-H), 1662 (C=O); Anal. Calcd. For C_13_H_12_ClN_3_O_2_ (277.71): 56.22% C, 4.36% H, 15.13% N; Found: 56.34% C, 4.38% H, 15.03% N.

*N-Benzyl-5-tert-butyl-6-chloropyrazine-2-carboxamide* (**9**). White crystalline compound. Yield: 61%; m.p. 69.7–71.3 °C; ^1^H-NMR (DMSO-*d_6_*) δ 9.35 (t, 1H, *J* = 6.4 Hz, NH), 9.04 (s. 1H, H3), 7.33–7.20 (m, 5H, H2′, H3′, H4′, H5′, H6′), 4.48 (d, 2H, *J* = 6.4 Hz, NCH_2_), 1.48 (s, 9H, CH_3_); ^13^C-NMR (DMSO-*d_6_*) δ 162.8, 162.0, 145.5, 142.4, 140.1, 139.3, 128.4, 127.6, 127.0, 42.6, 38.6, 28.2; IR (cm^−1^) 3399 (N-H), 1669 (C=O); Anal. Calcd. For C_16_H_18_ClN_3_O (303.79): 63.26% C, 5.97% H, 13.83% N; Found: 63.39% C, 6.13% H, 13.67% N.

*5-tert-Butyl-6-chloro-N-(3-trifluoromethylbenzyl)pyrazine-2-carboxamide* (**10**). White crystalline compound. Yield: 59%; m.p. 74.4–75.3 °C; ^1^H-NMR (CDCl_3_) δ 9.21 (s, 1H, H3), 7.98 (bs, 1H, NH), 7.60 (s, 1H, H2′), 7.58–7.45 (m, 3H, H4′, H5′, H6′), 4.72 (d, 2H, *J* = 6.4 Hz, NCH_2_), 1.53 (s, 9H, CH_3_); ^13^C-NMR (CDCl_3_) δ 164.5, 162.3, 145.9, 140.8, 140.2, 138.8, 131.2, 131.1 (q, *J* = 32.3 Hz), 129.3, 124.5 (q, *J* = 3.9 Hz), 124.4 (q, *J* = 3.9 Hz), 123.9 (q, *J* = 272.9 Hz), 42.9, 38.9, 28.3; IR (cm^−1^) 3373 (N-H), 1676 (C=O); Anal. Calcd. For C_17_H_17_ClF_3_N_3_O (371.78): 54.92% C, 4.61% H, 11.30% N; Found: 54.86% C, 4.53% H, 11.35% N.

*5-tert-Butyl-6-chloro-N-(4-chlorobenzyl)pyrazine-2-carboxamide* (**11**). White crystalline compound. Yield: 82%; m.p. 67.5–68.7 °C; ^1^H-NMR (CDCl_3_) δ 9.19 (s, 1H, H3), 7.91 (bs, 1H, NH), 7.39–7.19 (m, 4H, H2′, H3′, H5′, H6′), 4.62 (d, 2H, *J* = 6.3 Hz, NCH_2_), 1.52 (s, 9H, CH_3_); ^13^C-NMR (CDCl_3_) δ 164.4, 162.1, 140.9, 140.1, 136.2, 133.5, 131.5,129.2, 128.9, 42.7, 38.9, 28.3; IR (cm^−1^) 3376 (N-H), 1659 (C=O); Anal. Calcd. For C_16_H_17_Cl_2_N_3_O (338.23): 56.82% C, 5.07% H, 12.42% N; Found: 56.76% C, 5.18% H, 12.25% N.

*5-tert-Butyl-6-chloro-N-(4-methoxybenzyl)pyrazine-2-carboxamide* (**12**). White crystalline compound. Yield: 84%; m.p. 71.3–72.7 °C; ^1^H-NMR (CHCl_3_) δ 9.19 (s, 1H, H3), 7.84 (bs, 1H, NH), 7.31–7.25 (m, 2H, H2′, H6′), 6.91–6.85 (m, 2H, H3′, H5′), 4.59 (2H, d, *J* = 6.0 Hz), 3.80 (3H, s), 1.52 (9H, s); ^13^C NMR (CDCl_3_) δ 164.1, 161.9, 159.1, 141.1, 140.2, 140.1, 129.7, 129.3, 114.1, 55.3, 42.9, 38.8, 28.2; IR (cm^−1^) 3324 (N-H), 1673 (C=O); Anal. Calcd. For C_17_H_20_ClN_3_O_2_ (333.81): 61.17% C, 6.04% H, 15.13% N; Found: 61.23% C, 6.17% H, 15.01% N.

### 3.4. HPLC Lipophilicity Determination (Capacity Factor k/ Calculated Log k)

An Agilent Technologies 1200 SL liquid chromatography system equipped with a Diode-Array Detector SL G1315C, chromatographic pre-column ZORBAX XDB-C18 5 µm, 4 × 4 mm, Part No. 7995118-504 and column ZORBAX Eclipse XDB-C18 5 µm, 4.6 × 250 mm, Part No. 7995118-585 (Agilent Technologies Inc., Colorado Springs, CO, USA) were used. The separation process was controlled by Agilent ChemStation, version B.04.02 extended by spectral module (Agilent Technologies Inc.). A solution of MeOH (HPLC grade, 70%) with H_2_O (HPLC-Milli-Q Grade, 30%) was used as mobile phase. The total flow of the column was 1.0 mL/min, injection 20 µL, column temperature 30 °C. 210 nm as detection wavelength and 270 nm as monitor wavelength were chosen. The KI methanol solution was used for the dead time (T_D_) determination. Retention times (T_R_) of synthesized compounds were measured in minutes. The capacity factors *k* were calculated using Microsoft Excel according to formula *k* = (T_R_ − T_D_)/T_D_, where T_R_ is the retention time of the solute and T_D_ denotes the dead time obtained via an unretained analyte. Log *k*, calculated from the capacity factor *k*, is used as the lipophilicity index converted to log *P* scale. 

### 3.5. Lipophilicity Calculations

Log *P* (the logarithm of the partition coefficient for *n*-octanol/water) and Clog *P* (the logarithm of *n*-octanol/water partition coefficient *P* based on established chemical interactions) were calculated using the program CS ChemBioDraw Ultra version 12.0 (CambridgeSoft, Cambridge, MA, USA).

### 3.6. Biological Methods

#### 3.6.1. Evaluation of *In Vitro* Antimycobacterial Activity

Microdilution panel method. Antimycobacterial evaluation was shielded by Department of Clinical Microbiology, University Hospital and Faculty of Medicine in Hradec Králové, Charles University in Prague, Czech Republic. Four mycobacterial strains were used: *M. tuberculosis* H37Rv CNCTC My 331/88, *M. avium* CNCTC My 80/72, *M. avium* CNCTC My 152/73 and *M. kansasii* CNCTC My 235/80 (Czech National Collection of Type Cultures, National Institute of Public Health, Prague, Czech Republic). Tested compounds were dissolved in DMSO (to final concentrations 100, 50, 25, 12.5, 6.25, 3.125 and 1.563 µg/mL), diluted with Šula’s semisynthetic medium (Trios, Prague, Czech Republic) and placed into microdilution panel. Tested species were added in the form of suspension in isotonic saline solution. The final concentration of DMSO did not exceed 1% (v/v), this concentration of DMSO did not affect the growth of mycobacteria. The cultures were grown in Šula’s semisynthetic medium at pH 6.0 and 37 °C. The antimycobacterial activity was determined visually after 14 days (6 days for *M. kansasii*) of incubation as minimally inhibition concentration (MIC, µg/mL), *i.e.*, the lowest concentration of tested substance which inhibited the growth of mycobacteria.

#### 3.6.2. Evaluation of *In Vitro* Antifungal Activity

The Department of Medical and Biological Sciences at the Faculty of Pharmacy in Hradec Králové, Charles University in Prague, Czech Republic, performed the antifungal susceptibility assays, which was carried out using microdilution broth method [29,30]. Compounds were dissolved in DMSO and diluted in a twofold manner with RPMI 1640 medium with glutamine buffered to pH 7.0 (3-morpholinopropane-1-sulfonic acid). The final concentration of DMSO in the tested medium did not exceed 2.5% (v/v) of the total solution composition. Drug-free controls were included. Fluconazole was used as standard. The MICs were determined after 24 and 48 h, respectively after 72 and 120 h for *Trichophyton mentagrophytes* (TM), of static incubation in dark at 35 °C. Tested species: *Candida albicans* ATCC 44859, *C. tropicalis* 156, *C. krusei* E28, *C. glabrata* 20/I, *Trichosporon asahii* 1188, *Aspergillus fumigates* 231, *Absidia corymbifera* 272 and *Trichophyton mentagrophytes* 445 (TM).

#### 3.6.3. Study of the Inhibition of Oxygen Evolution rate in Spinach Chloroplasts

Chloroplasts were prepared from spinach (*Spinacia oleracea* L.) according to Masarovičová and Kráľová [31]. The inhibition of photosynthetic electron transport (PET) in spinach chloroplasts was determined spectrophotometrically (Genesys 6, Thermo Scientific, Madison, WI, USA) using an artificial electron acceptor 2,6-dichlorophenol-indophenol (DCPIP) according to Kráľová *et al.* [33] and the rate of photosynthetic electron transport (PET) was monitored as a photo-reduction of DCPIP. The measurements were carried out in a phosphate buffer (0.02 mol/L, pH 7.2) containing sucrose (0.4 mol/L), MgCl_2_ (0.005 mol/L) and NaCl (0.015 mol/L). The chlorophyll content was 30 mg/L in these experiments and the samples were irradiated (~100 W/m^2^) from a 10 cm distance with halogen lamp (250 W) using a 4 cm water filter to prevent warming of the samples (suspension temperature 22 °C). The studied compounds were dissolved in DMSO due to their limited water solubility. The applied DMSO concentration (up to 4%) did not affect the photochemical activity in spinach chloroplasts (PET). The inhibitory efficiency of the studied compounds was expressed as the IC_50_ values, *i.e.*, molar concentration of the compounds causing 50% decrease in the oxygen evolution relative to the untreated control. The comparable IC_50_ value for a selective herbicide 3-(3,4-dichlorophenyl)-1,1-dimethylurea (Diurone®, DCMU) was about 1.9 µmol/L [33]. 

#### 3.6.4. Study of Fluorescence of Chlorophyll *a* and Aromatic Amino Acids in Spinach Chloroplasts

The fluorescence emission spectra of chlorophyll *a* (Chl*a*) and aromatic amino acids in spinach chloroplasts were recorded on fluorescence spectrophotometer F-2000 (Hitachi, Tokyo, Japan) using excitation wavelength λ_ex_ = 436 nm for monitoring fluorescence of Chl*a* and λ_ex_ = 275 nm for monitoring fluorescence of aromatic amino acids, excitation slit 20 nm and emission slit 10 nm. The samples were kept in the dark 2 min before measuring. The phosphate buffer used for dilution of the chloroplast suspension was the same as described above. Due to low aqueous solubility the compounds were added to a chloroplast suspension in DMSO solution. The DMSO concentration in all samples was the same as in the control (10%). The chlorophyll concentration in chloroplast suspension was 10 mg/L.

## 4. Conclusions

A series of twelve binuclear pyrazinamide analogues containing -CONH-CH_2_- bridges was synthesized by the condensation of substituted pyrazine-2-carboxylic acid chlorides with the corresponding benzylamines. The final products were characterized by analytical data and evaluated for their *in vitro* antimycobacterial, antifungal, antibacterial and photosynthesis-inhibiting activity. Lipophilicity of the compounds was determined using RP-HPLC method and calculated using predicting program CS ChemBioDraw Ultra version 12.0. The obtained values were compared with each other and graphically expressed as dependence of calculated Clog *P* on log *k*. Compounds **8**, **10** and **12** had *in vitro* antimycobacterial activity against *M. tuberculosis* comparable with PZA (MIC = 6.25 µg/mL). More importantly, 5-*tert*-butyl-6-chloro-*N*-(4-methoxybenzyl)pyrazine-2-carboxamide (**12**) was active against tested MOTTs (*M. kansasii* and *M. avium*), which are unsusceptible to PZA. No clear dependence between lipophilicity and antimycobacterial activity has been found in this series. The highest antifungal activity (MIC = 15.6 µmol/L) against susceptible strain (*Trichophyton mentagrophytes*) was observed for 5-chloro-*N*-(3-trifluoromethylbenzyl)pyrazine-2-carboxamide (**2**), other compounds showed only weak or none *in vitro* antifungal activity. None of the studied compounds exhibited any significant activity against tested bacterial strains. 5-*tert*-butyl-6-chloro-*N*-benzylpyrazine-2-carboxamide (**9**, IC_50_ = 7.4 µmol/L). The most active compounds in inhibition of PET in spinach chloroplasts (*Spinacia oleracea* L.) were 5‑ *tert*-butyl-6-chloro-*N*-benzylpyrazine-2-carboxamide (**9**, IC_50_ = 7.4 µmol/L) and 5-*tert*-butyl-6-chloro-*N*-(4-chlorobenzyl)-pyrazine-2-carboxamide (**11**, IC_50_ = 13.4 µmol/L). Based on the obtained results it can be assumed, that in this series of pyrazinamide derivatives the antimycobacterial and antifungal activities did not depend directly on compound’s lipophilicity. On the other hand, PET-inhibiting activity increased linearly with compound’s lipophilicity. 
